# Rapid Decline in HCV Incidence among People Who Inject Drugs Associated with National Scale-Up in Coverage of a Combination of Harm Reduction Interventions

**DOI:** 10.1371/journal.pone.0104515

**Published:** 2014-08-11

**Authors:** Norah E. Palmateer, Avril Taylor, David J. Goldberg, Alison Munro, Celia Aitken, Samantha J. Shepherd, Georgina McAllister, Rory Gunson, Sharon J. Hutchinson

**Affiliations:** 1 Blood-borne Viruses and Sexually Transmitted Infections Section, Health Protection Scotland, Glasgow, United Kingdom; 2 Institute for Applied Social and Health Research, University of the West of Scotland, Paisley, United Kingdom; 3 West of Scotland Specialist Virology Centre, Glasgow Royal Infirmary, Glasgow, United Kingdom; 4 School of Health and Life Sciences, Glasgow Caledonian University, Glasgow, United Kingdom; Temple University School of Medicine, United States of America

## Abstract

**Background:**

Government policy has precipitated recent changes in the provision of harm reduction interventions – injecting equipment provision (IEP) and opiate substitution therapy (OST) – for people who inject drugs (PWID) in Scotland. We sought to examine the potential impact of these changes on hepatitis C virus (HCV) transmission among PWID.

**Methods and Findings:**

We used a framework to triangulate different types of evidence: ‘group-level/ecological’ and ‘individual-level’. Evidence was primarily generated from bio-behavioural cross-sectional surveys of PWID, undertaken during 2008-2012. Individuals in the window period (1–2 months) where the virus is present, but antibodies have not yet been formed, were considered to have recent infection. The survey data were supplemented with service data on the provision of injecting equipment and OST. Ecological analyses examined changes in intervention provision, self-reported intervention uptake, self-reported risk behaviour and HCV incidence; individual-level analyses investigated relationships within the pooled survey data. Nearly 8,000 PWID were recruited in the surveys. We observed a decline in HCV incidence, per 100 person-years, from 13.6 (95% CI: 8.1–20.1) in 2008–09 to 7.3 (3.0–12.9) in 2011–12; a period during which increases in the coverage of OST and IEP, and decreases in the frequency of injecting and sharing of injecting equipment, were observed. Individual-level evidence demonstrated that combined high coverage of needles/syringes and OST were associated with reduced risk of recent HCV in analyses that were unweighted (AOR 0.29, 95%CI 0.11–0.74) and weighted for frequency of injecting (AOR_w_ 0.05, 95%CI 0.01–0.18). We estimate the combination of harm reduction interventions may have averted 1400 new HCV infections during 2008–2012.

**Conclusions:**

This is the first study to demonstrate that impressive reductions in HCV incidence can be achieved among PWID over a relatively short time period through high coverage of a combination of interventions.

## Introduction

People who inject drugs (PWID) are at risk of contracting the hepatitis C virus (HCV) through the sharing of injecting equipment. Harm reduction interventions to prevent the transmission of HCV include opiate substitution therapy (OST), which aims to help PWID reduce their frequency of, or cease, injecting, and sterile injecting equipment provision (IEP), which aims to ensure that any injections that do take place are done with a clean set of equipment. Needles/syringes have long been implicated in HCV transmission; however, increasing evidence suggests that other injecting paraphernalia used in the drug preparation process (e.g. filters, spoons and water) may also contribute to transmission [1], [2].

Two reviews of the literature have highlighted that there is insufficient evidence demonstrating the effectiveness of certain harm reduction interventions – particularly IEP – on HCV transmission among PWID [3], [4]. A few studies have examined the impact of combining harm reduction interventions [5]–[7], but there remains a need to strengthen our understanding of the effectiveness of OST and IEP [8], [9]. Furthermore, previous studies of the impact of IEP on HCV incidence have focused solely on sterile needle/syringe provision (NSP); hitherto, no studies to date have directly examined the impact of providing non-needle/syringe injecting paraphernalia (primarily spoons/cookers and filters) in the prevention of HCV transmission [10].

In 2008, the Scottish Government launched the Hepatitis C Action Plan for Scotland (Phase II), with one of its three main aims to prevent HCV transmission among PWID. The main driver for change was the release of the National IEP Guidelines, which recommended [11] the provision of a set of new sterile injection equipment for every injection, and additional dedicated funding (£3 million per year) awarded to NHS Boards to enable improvement of services in accordance with these Guidelines. Contemporaneously to the Action Plan, the Scottish Government's drug and alcohol strategy, Road to Recovery [12], strived to improve treatment services for those dependent on opiates. In this paper, we seek to examine the potential impact of these changes on HCV transmission among PWID: specifically, the aim is to determine the association between harm reduction interventions (IEP and OST) and incident HCV infection among PWID in Scotland during 2008 to 2012. To do this, we examine the results of three national cross-sectional surveys of PWID, as well as data on IEP and OST provision. These data allow us to examine trends in uptake of harm reduction interventions, risk behaviour and HCV incidence. Additionally, the pooling of these surveys generates the largest sample to have explored HCV transmission in relation to the combined effects of NSP and OST.

## Methods

**Figure 1:**
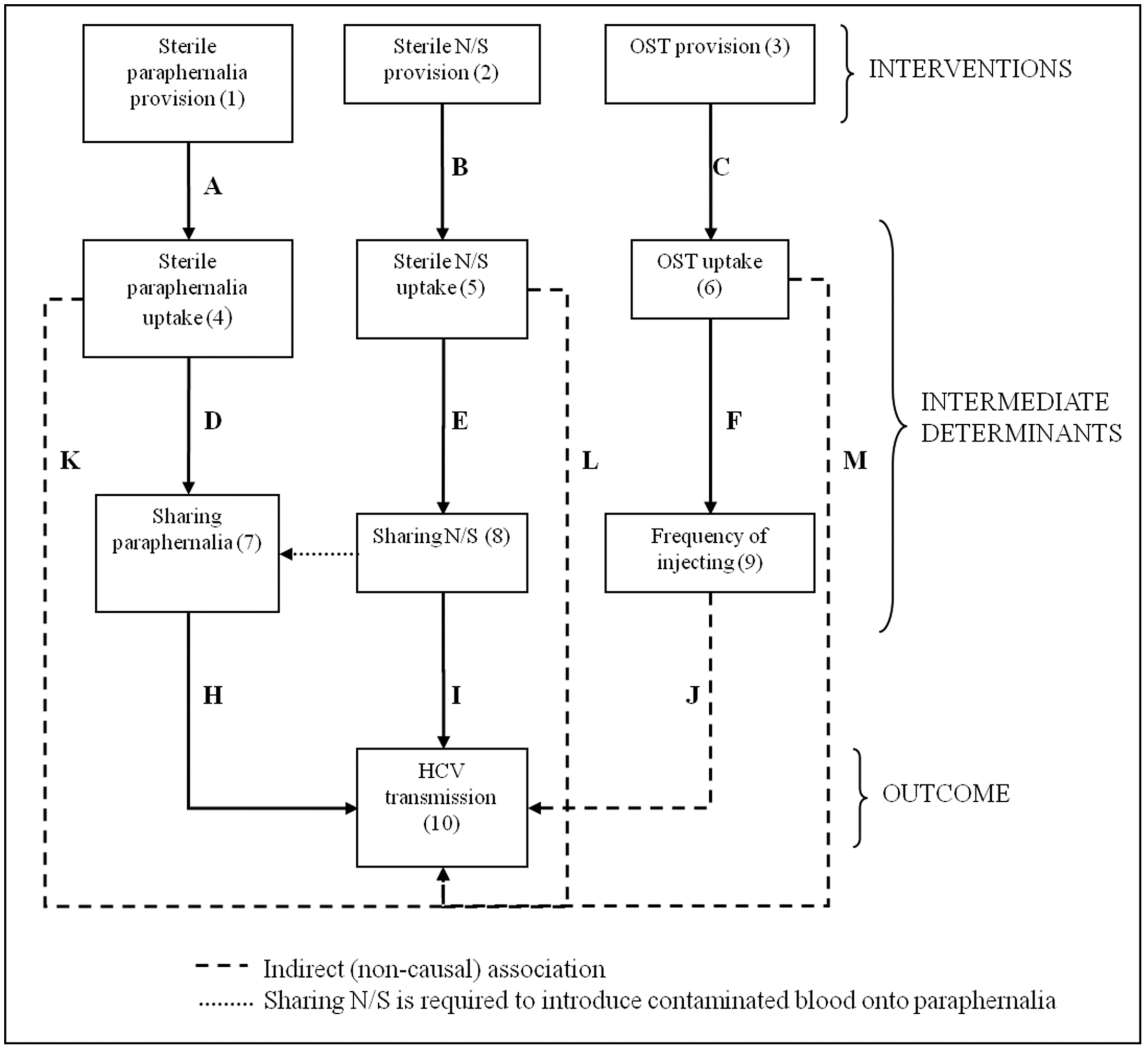
Analytical framework of potential associations between harm reduction interventions and HCV transmission. The arrows represent investigated associations: relationships A through C are group-level and relationships D through M are individual-level.

The difficulties in undertaking what would traditionally be considered ‘robust’ study designs to investigate public health interventions have been well documented [13]–[15]. Nevertheless, some common themes that have emerged from such evaluations, in relation to causal attribution, are (i) the importance of understanding the processes/pathways between intervention(s) and outcome(s) [16] and (ii) the combination of evidence generated from different non-experimental study designs [17]. The analytical approach applied here borrows from these themes: first, to understand pathways, an analytical framework was produced to guide the analysis (Figure 1). The objective was to describe each of the elements of the framework, as well as the relationships between them, to build an overall picture of the potential mechanisms between provision of interventions and HCV transmission. Secondly, in relation to combining evidence from different study designs, the approach taken here triangulates evidence from ecological and individual-level approaches (described further below). All of the information was collated and summarised in a table, as a means of capturing the evidence for the framework. Unless otherwise indicated, most of this was derived from analysis of the surveys described further below.

### Data collection

Ethical approval was obtained from the West of Scotland Research Ethics Service. Three sweeps of a series of cross-sectional surveys of PWID were undertaken in June 2008–June 2009, January–November 2010, and March 2011–March 2012. These voluntary anonymous surveys are collectively called the Needle Exchange Surveillance Initiative (NESI); the methods have been described in detail previously [6], [18]. Briefly, PWID were recruited at sites that provide sterile injecting equipment (and often methadone) across mainland Scotland. People who had ever injected drugs were eligible to participate, although the majority (approximately 80%) had injected in the last six months. The interviewer informed potential participants of the nature of the study by giving them an information sheet outlining the purpose of the research and what it would mean to them if they agreed to participate. Individuals who agreed to participate were then asked to sign (initials only, to maintain anonymity) a consent form. Subsequently, participants completed an interviewer-administered questionnaire and provided a blood spot for laboratory testing.

Data on the provision of OST and IEP in Scotland were obtained from routine reports published in the grey literature [19], [20].

### Laboratory methods

The dried blood spots (DBS) were eluted and tested in a modification of the Ortho Save 3.0 EIA, which has 99% sensitivity and 100% specificity for the detection of HCV antibody (anti-HCV) on DBS [21]. The assay has been adapted further to use two 3 mm discs punched from DBS and eluted in 200 µl of PBS/0.05% tween. The optical densities of <0.4, 0.4–0.79 and ≥0.8 were classified as negative, equivocal and positive for anti-HCV, respectively. HCV-RNA testing was undertaken on anti-HCV negative samples using an ‘in house’ PCR assay [22]; the sensitivity and specificity of this assay on DBS are 100% and 96%, respectively.

## Analysis

### Outcome measure

We defined recent HCV infections as individuals who were anti-HCV negative and HCV-RNA positive. Incidence was derived using the formula 
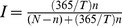
where T is the estimated duration of the window period (where the virus is detectable but prior to the formation of antibodies), n is the number of recent infections and N is the number of susceptibles (i.e. anti-HCV negatives) [6], [23]. An estimate of the duration of the window period (mean 51 days; variance 56 days), was derived from the literature [24]. Ninety-five percent confidence intervals for the incidence rates in each survey were generated by bootstrapping: (i) sampling 1,000 values from each of the binomial and normal distributions relating to the number of recent infections and the window period, respectively; (ii) using the sampled values from (i) in the formula to generate a distribution for the incidence rates; and (iii) taking the 2.5th and 97.5th percentile values from (ii) to generate the lower and upper confidence limits.

In order to validate the derived HCV incidence estimates, we also examined HCV prevalence among those who commenced injecting within the last 12 months, based on the assumption that HCV infection will have been acquired since initiation of injecting.

### Intervention measures

Variables categorising participants into high and low ‘coverage’ of each injecting equipment item were created by dividing the self-reported number of items (needles/syringes, spoons, filters, or water ampoules) obtained in the last six months, by the self-reported number of injections undertaken in the last six months. These variables will henceforth be referred to as ‘self-report IEP coverage’. The threshold for high coverage (≥200%) was chosen on the basis of sensitivity analyses. To reduce collinearity, the spoon and filter coverage variables were further combined into a single variable called paraphernalia coverage, such that those who reported high coverage of both spoons and filters were classified as having high paraphernalia coverage, with the remaining falling into the low category; water coverage was not considered since self-reported sharing of water was not found to be associated with recent HCV infection in our analyses [2]. Participants were also categorised by whether they reported being on OST at the time of the survey (yes/no). OST refers to methadone maintenance as this is, overwhelmingly, the primary pharmacological treatment used to treat opiate addiction in Scotland. Those who reported not injecting in the last 6 months and no uptake of any interventions were excluded (n = 157, 2.2% of the pooled sample).

For the purposes of comparing trends over time at the group-level, an additional coverage measure was calculated by dividing published numbers of injecting equipment items distributed [19] by estimates of the total number of injections annually among Scottish PWID (the latter generated by multiplying the estimated mean annual number of injections per PWID from NESI with estimates of the size of the injecting population [25]). This will henceforth be referred to as ‘service provision IEP coverage’.

### Group-level/ecological analysis

We use ‘group-level’ to refer to the statistics that describe the interventions, intermediate determinants, and outcomes (i.e. the boxes in Figure 1), and any changes therein. The following were compared across the three surveys: (i) harm reduction intervention uptake, (ii) risk behaviour, (iii) HCV prevalence and iv) HCV incidence. Statistical significance was assessed using either the Mantel-Haenszel test for trend for categorical variables or ANOVA for continuous variables. In the ecological analyses, ‘adequate’ coverage was defined as at least one sterile item per injection (i.e.≥100%). In terms of risk behaviour, ‘sharing’ was defined as the use of an item of injecting equipment after it had previously been used by someone else.

Respondents who had participated multiple times were identified from within each survey: 115 (1.4%), 147 (1.8%) and 40 (0.5%) duplicates were excluded from the 2008–09, 2010 and 2011–12 surveys, respectively.

### Individual-level analysis

Logistic regression was used to investigate the associations D through M (Figure 1). Confounding variables that were considered included survey year, gender, age, homelessness and stimulant injection (in the last 6 months), time since onset of injecting, imprisonment (ever) and alcohol consumption (last 12 months). A backwards stepwise approach to model building was applied; however, certain variables that had been identified *a priori* (e.g. survey year) were included regardless of significance. Analyses were undertaken in SPSS version 21.

Five multivariable models were built to examine the association with recent HCV of (self-reported): (i) N/S coverage, (ii) paraphernalia coverage, (iii) OST, (iv) N/S coverage and OST, and (v) all three interventions combined. Weighted models were subsequently run in Stata 9. Sampling weights were set to be equal to the number of times that a respondent reported injecting in the six months prior; thus, observations from individuals who reported injecting more frequently counted more heavily in the analysis. Sampling weights were increased by one, such that individuals who reported not injecting in the last 6 months were included (with a weight of 1).

### New infections and infections averted

The number of new chronic infections was estimated for each calendar year (2008–2012) by combining the derived incidence rates with published estimates of the size of the PWID population [25], estimates of anti-HCV prevalence from NESI, and published estimates of the proportion of HCV-infected individuals who develop chronic infection (26% on average were assumed to clear acute infection) [26]. It was assumed that the size of the PWID population remained stable during this period. The method for generating a distribution of values for the incidence rates has been described above. Additionally, posterior distributions for the size of the PWID population, the proportion anti-HCV negative, and the proportion that develop chronic infection were generated. One thousand values were sampled from each of the latter distributions. The sampled values for the number of PWID were multiplied by those for the proportion anti-HCV negative, to generate a distribution for the number of susceptible PWID (i.e. anti-HCV negatives). The latter were then multiplied by the sampled incidence values and the sampled values for the proportion developing chronic infection, to generate a distribution (and 95% CI, as previously described) for the number of new chronic HCV infections. An estimate of the number of HCV infections (all and chronic) potentially averted by harm reduction interventions over the period 2008–2012 was calculated by subtracting the sum total of the calculated yearly estimates from that which would have been observed assuming the number of infections in 2008 had remained constant.

## Results

The average response rate across the surveys was 64%. The gender distribution was nearly identical between the pooled survey participants (72% male) and those who refused to participate (73% male); however, those who refused were slightly younger on average (31 years) as compared with participants (34 years).

Table 1 summarises the demographic characteristics of the study population by survey sweep. Significant differences in several variables were noted between the surveys: mean age increased from 33.6 in 2008–09 to 35.3 in 2011–12 (*P*<0.001), as did mean time since onset of injecting, with respective figures of 10.5 and 11.6 years (*P*<0.001). The proportion of respondents who reported homelessness in the last 6 months decreased slightly from 27% to 22% (*P*<0.001), as did the proportion who reported injecting stimulants (23% to 15%, *P*<0.001).

**Table 1:** Demographic and other characteristics of study population, by survey.

	2008–09 (N = 2,629)	2010 (N = 3,168)	2011–12 (N = 2,154)	X^2^ test (trend) or ANOVA	*P* value
Male gender	72%	72%	73%	0.086	0.770
Mean age in years (SD)	33.6 (7.1)	34.6 (7.3)	35.3 (6.9)	F = 35.465	<0.001
Aged <25 years	14%	12%	9%	37.000	<0.001
Homeless in last 6 months	27%	22%	22%	21.370	<0.001
Injected stimulants in last 6 months^a^	23%	13%	15%	45.416	<0.001
Ever in prison	59%	61%	61%	2.213	0.137
Excessive alcohol consumption^b^ (last 12 months)	27%	24%	26%	0.572	0.449
Mean time since onset of injecting in years (SD)	10.5 (7.4)	11.2 (7.7)	11.6 (7.4)	F = 15.247	<0.001
Commenced injecting within the last 5 years	26%	24%	21%	11.970	0.001

ANOVA =  analysis of variance; SD =  standard deviation.

a Among those who reported injecting in the last six months.

b Defined as >14 units/week for women and >21 units/week for men.

A summary of the evidence for each of the elements of the framework is provided in Table 2, and discussed in more detail below.

**Table 2:** Summary of evidence for the analytical framework.

Type of evidence	Category	Reference to Fig.1	Description	N/S	OST	Paraphernalia
Group-level	Intervention	1, 2, 3	Changes in provision of intervention over time	Minor fluctuations but relatively stable number of N/S distributed at approximately 4.7 million annually (Table S1)	More or less stable, if very slight increase, in number of methadone mixture prescriptions dispensed (Table S1)	Several-fold increase in number of spoons and filters distributed, up to approximately 2.5 million each annually (Table S1)
	Intermediate determinant	4, 5, 6	Changes in uptake of intervention over time	Decline in self-reported uptake of N/S but, given the concurrent declines in frequency of injecting, this translates into either a relatively stable or increasing proportion of PWID with adequate (≥100%) N/S coverage, depending on which measure of coverage is used (Table 3)	Increase in those reporting currently being on OST (Table 3)	Increase in reported numbers of spoons and filters obtained, as well as increase in reported proportion with high filter and spoon coverage – reaching almost equal coverage to that of N/S (Table 3)
	Intermediate determinant	7, 8, 9	Changes in risk behaviour over time	Reduction in proportion who reported sharing N/S in last six months (Table 3)	Decline in the proportion that reported injecting daily or more frequently in the last six months (Table 3)	Decline, for both spoons and filters, in proportion who reported sharing in last six months (Table 3)
	Outcome	10	Changes in HCV incidence over time	Decline in estimated HCV incidence between 2008–09 and 2011–12; validated by a parallel decline in the prevalence of anti-HCV among those who commenced injecting within the last 12 months (Table 3)		
Individual-level	Relationship	D, E, F	Association between uptake of intervention and concomitant risk behaviour	Approx 55% reduction in the odds of having shared N/S in the last 6 months among those with high N/S coverage (Table S3)	Nearly 80% reduction in the risk of injecting daily or more frequently in the last six months associated with current uptake of OST (Table S6)	∼35% reduction in the odds of having shared spoons in the last 6 months among those with high spoon coverage. High filter coverage associated with ∼20% reduction in odds of sharing filters (Tables S4 and S5)
	Relationship	H, I, J	Association between risk behaviour and HCV incidence	Approx 7-fold increased risk of recent HCV infection associated with sharing N/S in the last 6 months [2] a	No direct association between frequency of injecting and recent HCV infection (Table S7); but, injecting at least daily is associated with 3-fold increased risk of sharing (each of) N/S, spoons, and filters (Tables S8 to S10)	Approx 3-fold increased risk of recent HCV infection associated with sharing spoons OR filters in the last 6 months [2] a
	Relationship	K, L, M	Association between uptake of intervention and HCV incidence	Strong association between high coverage N/S and recent HCV in unweighted analyses (AOR 0.4, 95% CI 0.2–0.8, *P* = 0.014) and weighted analyses (AOR_w_ 0.1, 95% CI 0.04–0.5, *P* = 0.002) (Table 4)	No evidence for an association between current OST and recent HCV, in either weighted or unweighted analyses (Table 4)	High paraphernalia coverage associated with recent HCV in unweighted analyses (AOR_w_ 0.4, 95% CI 0.1–1.1, *P* = 0.08) but more so in weighted model (AOR_w_ 0.1, 95% CI 0.03–0.4, *P* = 0.002) (Table 4)
				Evidence of a strong association between the combined effects of N/S and OST and a reduced risk of recent HCV (AOR 0.3, 95% CI 0.1–0.8, *P* = 0.02; and AOR_w_ 0.05, 95% CI 0.01–0.19, *P*<0.001). Combined effects of N/S, OST, and paraphernalia (AOR 0.3, 95% CI 0.1–1.1, *P* = 0.064 and AOR_w_ 0.07, 95% CI 0.01–0.37, *P* = 0.002) are not substantially different from that of N/S and OST combined. (Table 4)		

(A)OR =  (adjusted) odds ratio; N/S =  needles/syringes; OST =  opiate substitution therapy; PWID =  people who inject drugs.

a Note that this study found an AOR for sharing needles/syringes (+/− paraphernalia) of 6.7 (95% CI 2.6–17.1) and an AOR for sharing paraphernalia only of 3.0 (95% CI 1.2–7.5). It was not possible to separate the effects of needles/syringes due to few individuals who reported sharing only needles/syringes. It is, however, assumed that the potential AOR for needles/syringes could be even higher than that detected for needles/syringes +/− paraphernalia.

### Group-level analyses

Firstly, the major changes in provision of interventions (Boxes 1,2, and 3 in Figure 1) that took place were increases in the provision of filters and spoons by 6-fold and 4-fold, respectively, between 2008/09 and 2009/10 financial years (Table S1). By contrast, provision of N/S remained approximately stable over the period, hovering at around 4.7 million distributed annually, albeit with minor relative fluctuations. The number of methadone prescriptions dispensed in Scotland increased only slightly up until 2010/11 and then declined by 4% in 2011/12.

In terms of harm reduction intervention uptake (boxes 4 through 6 in Figure 1), the proportion who reported currently receiving OST increased from 50% to 64% (*P*<0.001) between 2008–09 and 2011–12 (Table 3). Despite the slight decline in the median number of sterile needles/syringes obtained (based on self-report survey data), the proportion of individuals with adequate N/S coverage was more or less stable (75%–79%) due to simultaneous declines in frequency of injecting. The proportion of individuals with adequate coverage of filters and spoons increased between 2008–09 and 2011–12 (from 24% to 69% and from 20% to 70%, respectively, both *P*<0.001). Using the measure of IEP coverage based on service provision data, the proportion with adequate N/S, filter, and spoon coverage increased from 53% to 74%, 4% to 40%, and 6% to 39%, respectively. These changes were mostly attributable to declines in the frequency of injecting (Table S2).

**Table 3:** Group-level analyses: risk behaviour and harm reduction intervention uptake of study sample, by survey.

		2008–09 (N = 2,629)	2010 (N = 3,168)	2011–12 (N = 2,154)	Test for difference between years^a^	P value
***(i) Intervention uptake (last 6 months)***						
Currently on OST	All PWID^b^^,^ c	50%	60%	64%	48.442	<0.001
PWID, current^b^^,^ c		49%	60%	64%	50.564	<0.001
Median no. N/S obtained in typical week in last 6 months (IQR)^c^		15 (25)	10 (17)	10 (18)	113.493	<0.001
Median no. filters obtained in typical week in last 6 months (IQR)^c^		0 (8)	8 (20)	10 (19)	794.167	<0.001
Median no. spoons obtained in typical week in last 6 months (IQR)^c^		0 (5)	7.5 (19)	10 (18)	1026.638	<0.001
Self-report IEP Coverage^d^						
Proportion with adequate N/S coverage (≥100%)^c^		75%	79%	77%	3.271	0.071
Proportion with adequate filter coverage (≥100%)^c^		24%	58%	69%	817.385	<0.001
Proportion with adequate spoon coverage (≥100%)^c^		20%	58%	70%	972.267	<0.001
Service provision IEP Coverage^d^						
Proportion with adequate N/S coverage (≥100%)		53%	62%	74%		
Proportion with adequate filter coverage (≥100%)		4%	34%	40%		
Proportion with adequate spoon coverage (≥100%)		6%	33%	39%		
***(ii) Risk behaviour (last 6 months)***						
Injected at least daily^c^		63%	54%	49%	73.712	<0.001
Shared N/S^c^		15%	11%	8%	51.497	<0.001
Mean no. times shared N/S (SD)^c^		0.89 (4.0)	0.69 (4.2)	0.46 (4.0)	F = 5.357	0.005
Mean no. times shared N/S among those who shared (SD)^c^		5.9 (8.7)	6.5 (11.4)	6.0 (13.0)	F = 0.209	0.812
Reused own needle/syringe^c^		64%	59%	45%	131.952	<0.001
Shared spoons^c^		42%	33%	20%	217.652	<0.001
Shared filters^c^		33%	28%	17%	123.088	<0.001
Shared water^c^		31%	29%	21%	48.740	<0.001

a X^2^ test for trend for proportions, Kruskal-Wallis test for medians, ANOVA test for means.

b Among those who cited needle exchange as the reason for visiting the service on the day of recruitment.

c Among those who reported injecting in the last 6 months.

d See methods for details. P-values have not been calculated for the second measure of IEP coverage – because of the large numbers, even clinically insignificant changes would be statistically significant.

The proportion of respondents reporting various risk behaviours in the last six months declined across the surveys (Table 3): injecting daily or more frequently (from 63% to 49%, *P*<0.001), sharing needles/syringes (from 15% to 8%, *P*<0.001), reusing one's own needles/syringes (64% to 45%, *P*<0.001), sharing spoons (42% to 20%, *P*<0.001), sharing filters (33% to 17%, *P*<0.001), and sharing water (31% to 21%, *P*<0.001).

**Figure 2:**
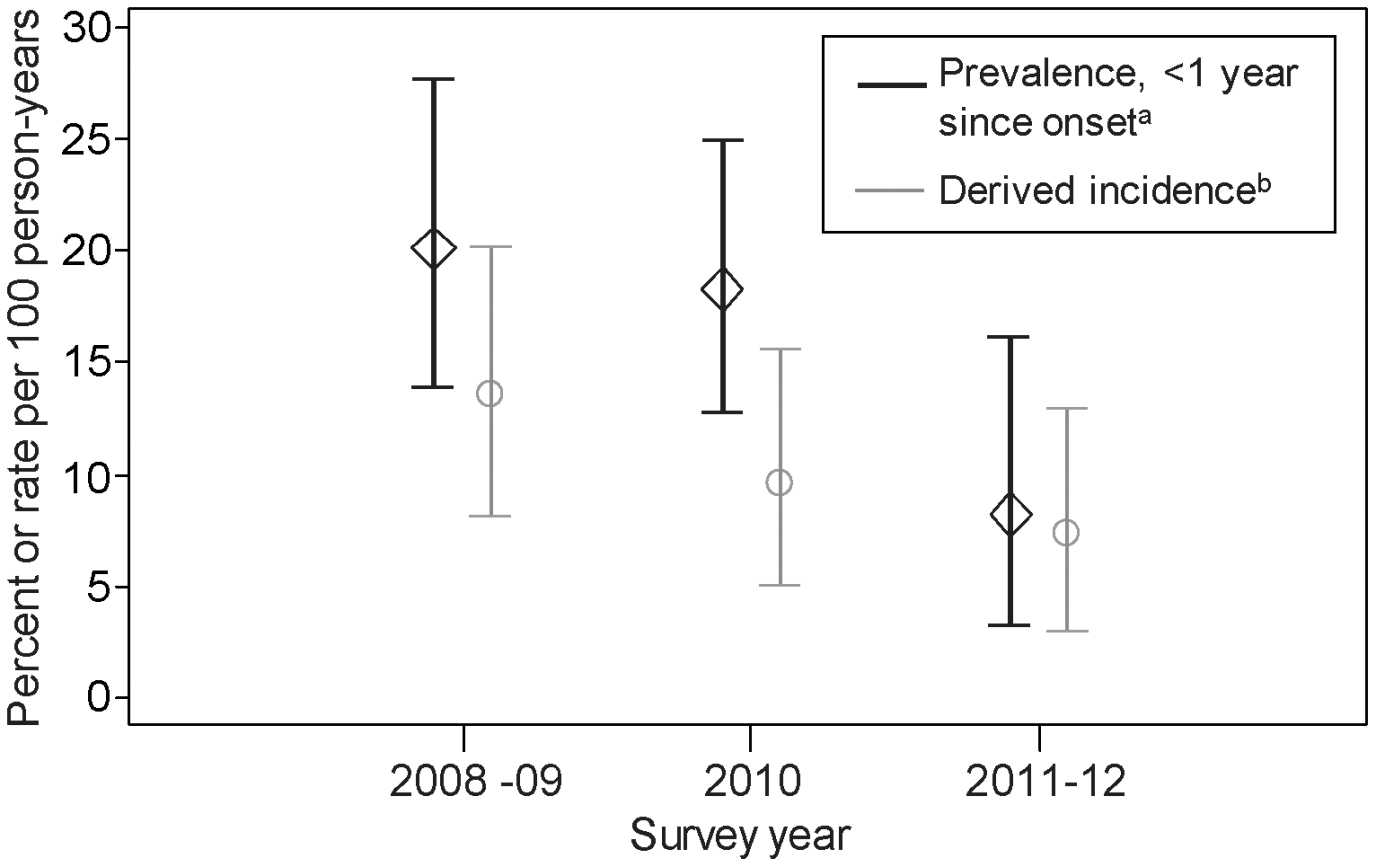
Prevalence (among recent onset injectors) and derived incidence of HCV among people who inject drugs in Scotland, 2008 to 2012. The diamonds/circles represent the point estimate and the bars represent the upper and lower 95% confidence intervals. ^a^anti-HCV prevalence among those who commenced injecting within the past 12 months. ^b^Determined by applying the estimated pre-seroconversion window period to the observed number of anti-HCV negative and HCV-RNA positive individuals (see methods for details). Restricted to those who had injected in the last 6 months.

A total of 53 recent infections were detected among 3,459 susceptible (i.e. anti-HCV negative) individuals. The proportion of recent HCV infections decreased from 2.1% (95% CI: 1.3%–3.3%) in 2008–09 to 0.9% (95% CI: 0.4%–1.7%) in 2011–12 (X^2^ test for trend: *P* = 0.02) (Table 4). The derived incidence rates per 100 person-years (among PWID, current) reduced from 13.6 (95% CI 8.1–20.1) in 2008–09, to 7.3 (3.0–12.9) in 2011–12. HCV prevalence among those who commenced injecting within the last year was comparable to the derived incidence rates for the respective years (Figure 2), declining from 20.1% (95% CI 13.9%–27.6%) in 2008–09 to 8.2% (95% CI 3.4%–16.2%) (*P* = 0.03).

**Table 4:** Group-level analyses: HCV prevalence and incidence, by survey.

	2008–09	2010	2011–12	X^2^ test for trend	P value
***(i) HCV prevalence***	(n = 2,629)	(n = 3,168)	(n = 2,154)		
HCV prevalence^a^ (95% CI)	54% (52–55%)	56% (54–58%)	53% (51–55%)	0.004	0.951
***(ii) HCV incidence***					
	(n = 144)	(n = 169)	(n = 85)		
HCV prevalence among those injecting <1 yr (95% CI)	20.1% (13.9–27.6%)	18.3% (12.8–25.0%)	8.2% (3.4–16.2%)	4.711	0.030
	(n = 1,140)	(n = 1,323)	(n = 996)		
Recent HCV infection (all PWID)^b^ (95% CI)	2.1% (1.4–3.1%)	1.5% (0.9–2.3%)	0.9% (0.4–1.7%)	5.092	0.024
	(n = 933)	(n = 1,024)	(n = 831)		
Recent HCV infection (PWID, current)^b^^,^ c (95% CI)	2.1% (1.3–3.3%)	1.5% (0.8–2.4%)	1.1% (0.5–2.0%)	3.224	0.073
Derived HCV incidence per 100 PY (all PWID)	13.3 (8.4–19.8)	9.9 (5.5–14.8)	6.1 (2.5–11.1)	-	-
Derived HCV incidence per 100 PY (PWID, current)	13.6 (8.1–20.1)	9.6 (5.1–15.5)	7.3 (3.0–12.9)	-	-

CI =  confidence interval; PWID =  people who inject drugs.

a Numerator includes anti-HCV positives and weak reactives; the denominator is all PWID.

b Recent infection is defined as anti-HCV negative and HCV-RNA positive; the denominator is all anti-HCV negative PWID.

c Among those who reported injecting in the last 6 months.

### Individual-level analyses

The individual-level associations between uptake of the interventions and injecting risk behaviour (relationships D, E & F) are presented in Tables S3, S4, S5, and S6 and summarised in Table 2: high coverage (≥200%) of N/S, spoons, and filters were significantly associated with approximately 55%, 35% and 20% reductions, respectively, in the odds of having shared these items in the last six months. Currently being on OST was associated with a nearly 80% reduction in the odds of injecting daily or more frequently in the last six months.

With regard to the associations between sharing injecting equipment and recent HCV infection (relationships H and I), we estimated the odds of recent HCV infection to be 7-fold and 3-fold for sharing needles/syringes and paraphernalia in the last six months, respectively, as compared with no sharing [2]. The analysis of the association between frequency of injecting and recent HCV infection (relationship J) is presented in Table S7: it was not statistically significant after adjustment for potential confounders (survey year, homelessness, stimulant injection, time since onset of injecting and alcohol consumption). Since this is an indirect association, we also examined and showed that the risk of sharing either needles/syringes, spoons, or filters, was 3 times higher among those who injected daily or more frequently as compared to those who did not (Tables S8, S9 and S10).

Table 5 presents the associations between uptake of harm reduction interventions and recent HCV infection (relationships K, L and M in Figure 1). The findings indicated that individuals with high N/S or high paraphernalia coverage had lower proportions of recent HCV infection (0.9% and 0.7%, respectively) as compared with those on low coverage of these interventions (2.4% and 2.0%, respectively). Individuals on OST at the time of survey had a lower proportion recently infected (1.3%) as compared with those not on OST (2.5%). The effect of the weighting by injecting frequency was generally to increase the differences in incidence between the high and low coverage groups.

**Table 5:** Individual-level analyses: unweighted and weighted models of the association between self-reported uptake of harm reduction interventions and recent HCV infection^a^.

					Univariable		Multivariable^b^		Weighted multivariable^b^	
Model	Intervention	Categories	Recent infection %	Weighted recent infection %	OR (95% CI)	*P* value	AOR (95% CI)	*P* value	AOR_w_ (95% CI)	*P* value
							(n = 2,481)		(n = 2,481)	
(i)	N/S coverage^c^	Low	2.4	2.9	1		1		1	
		High	0.9	0.4	0.36 (0.17–0.75)	0.006	0.39 (0.19–0.83)	0.014	0.14 (0.04–0.48)	0.002
							(n = 2,481)		(n = 2,481)	
(ii)	Paraphernalia coverage^c^^,^ d	Low	2.0	2.7	1		1		1	
		High	0.7	0.2	0.33 (0.12–0.94)	0.037	0.39 (0.14–1.12)	0.081	0.11 (0.03–0.44)	0.002
							(n = 3,008)		(n = 3,008)	
(iii)	OST	Not current	2.5	3.0	1		1		1	
		Current	1.3	1.3	0.51 (0.29–0.90)	0.020	0.63 (0.35–1.12)	0.111	0.52 (0.23–1.18)	0.119
							(n = 2,993)		(n = 2,993)	
(iv)	N/S and OST combined^e^	Low N/S, no OST	3.4	3.8	1		1		1	
		Low N/S, OST	1.6	2.0	0.48 (0.24–0.95)	0.035	0.55 (0.27–1.11)	0.093	0.59 (0.26–1.35)	0.209
		High N/S, no OST	0.9	0.7	0.26 (0.08–0.88)	0.030	0.28 (0.08–0.96)	0.043	0.18 (0.04–0.87)	0.034
		High N/S, OST	0.8	0.1	0.24 (0.10–0.60)	0.002	0.29 (0.11–0.74)	0.009	0.05 (0.01–0.18)	<0.001
		Did not inject, OST	1.4	1.4	0.39 (0.17–0.94)	0.035	0.62 (0.24–1.59)	0.324	0.61 (0.21–1.80)	0.370
							(n = 2,992)		(n = 2,992)	
(v)	N/S, paraphernalia and OST combined^e^	Low N/S, low para, no OST	3.5	3.9	1		1		1	
		Low N/S, low para, OST	1.7	2.0	0.48 (0.24–0.96)	0.037	0.55 (0.27–1.11)	0.095	0.58 (0.25–1.34)	0.202
		High N/S, low para, no OST	1.0	0.8	0.27 (0.06–1.15)	0.076	0.29 (0.07–1.26)	0.098	0.18 (0.03–1.34)	0.095
		High N/S, low para, OST	0.9	0.1	0.26 (0.08–0.89)	0.031	0.28 (0.08–0.98)	0.046	0.02 (0.01–0.09)	<0.001
		High N/S, high para, no OST	0.8	0.5	0.22 (0.03–1.65)	0.141	0.25 (0.03–1.90)	0.180	0.16 (0.02–1.25)	0.081
		High N/S, high para, OST	0.8	0.2	0.21 (0.06–0.71)	0.012	0.28 (0.08–0.97)	0.044	0.07 (0.01–0.35)	0.001
		Did not inject, OST	1.4	1.4	0.38 (0.16–0.90)	0.028	0.60 (0.24–1.54)	0.292	0.59 (0.20–1.75)	0.343

(A)OR =  (adjusted) odds ratio; CI =  confidence interval; OST =  opiate substitution therapy; N/S =  needle/syringe; para  =  paraphernalia.

a Where a recent infection is defined as anti-HCV negative and HCV-RNA positive.

b All models adjusted for survey year, homelessness in last 6 months, stimulant injection in last 6 months, time since onset of injecting.

c Restricted to those who reported injecting in the last 6 months; see methods for definition of high and low coverage.

d Paraphernalia refers to spoons and filters.

e Where ‘no OST/OST’ refers to no current/current receipt of OST, ‘low’ refers to low coverage, and ‘high’ refers to high coverage.

In multivariable unweighted analyses, both high N/S and paraphernalia coverage were associated with reduced risk of recent HCV (AOR 0.39, 95% CI 0.19–0.83, *P* = 0.014 and AOR 0.39, 95% CI 0.14–1.12, *P* = 0.081, respectively) relative to those with low coverage. In weighted analyses, both adjusted odds ratios moved farther away from null (AOR_w_ 0.14, 95% CI 0.04–0.48, *P* = 0.002 for N/S coverage and AOR_w_ 0.11, 95% CI 0.03–0.44, *P* = 0.002 for paraphernalia coverage). Being on OST at the time of the survey was not statistically associated with recent infection in either unweighted or weighted analyses (AOR 0.63, 95% CI 0.35–1.12; and AOR_w_ 0.52, 95% CI 0.23–1.18, respectively).

Model (iv) examined the combined effects of N/S coverage and OST. With the exception of those who did not inject in the last six months, there was a general downward gradient in incidence with increasing coverage of interventions, and this trend was more apparent in the weighted incidence. In the unweighted analyses, those with high N/S coverage had lower odds of recent infection, whether also on OST or not (AOR 0.28, 95% CI 0.08–0.96 and AOR 0.29, 95% CI 0.11–0.74). This was also the case for the weighted analyses, although there was a slight difference between the effect sizes, with those on high N/S coverage *and* current OST exhibiting a greater reduction in risk (AOR_w_ 0.05, 95% CI 0.01–0.18) as compared to those on high N/S and no OST (AOR_w_ 0.18, 95% CI 0.04–0.87).

**Figure 3:**
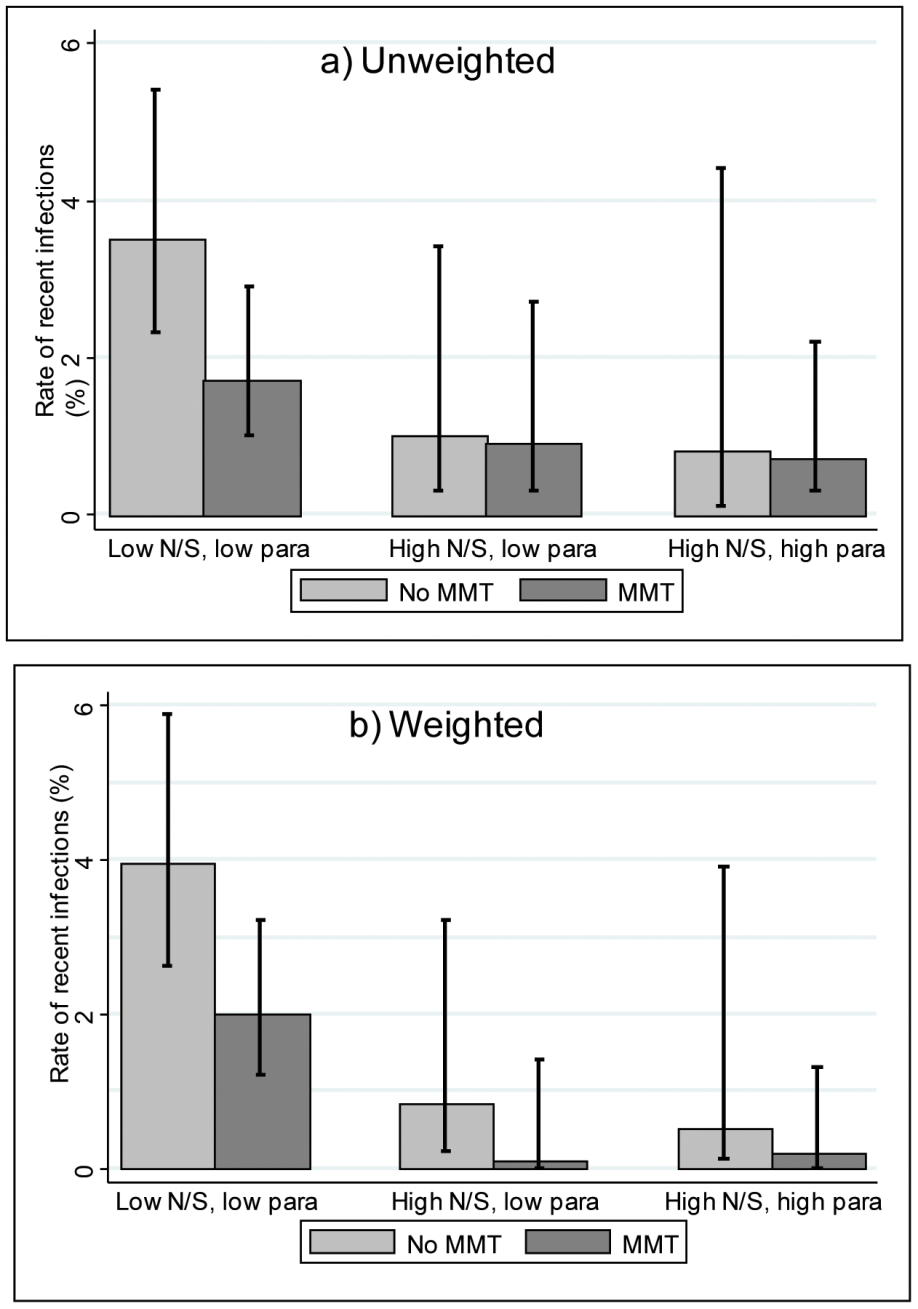
a) Unweighted and b) weighted HCV incidence among PWID in Scotland from pooled survey data (2008 to 2012), by harm reduction intervention uptake. 95% confidence intervals are indicated by the black bars.

Figure 3 presents proportions recently infected with HCV for the different strata of model (v). The highest incidence of HCV (3.5% unweighted and 3.9% weighted) was among the baseline group of those on the lowest coverage of all three interventions. Among PWID with low coverage of both needles/syringes and paraphernalia, the results were suggestive of a reduction in risk of approximately 40% for those on current OST (this can be also seen by the difference in the height of the two left-hand bars in Figure 3a and 3b); althoughit was not statistically significant after adjustment (AOR 0.55, 95% CI 0.27–1.11; AOR_w_ 0.58, 95% CI 0.25–1.34). There were no recent infections in the ‘low N/S, high para’ groups, due to very small numbers in these groups, and therefore it was not possible to calculate odds ratios. Moving from low to high N/S coverage was associated with lower HCV incidence, although the larger difference was seen among those not on OST (3.5% to 1.0% unweighted; 3.9% to 0.8% weighted) as compared to those on OST (1.7% to 0.9% unweighted; 2.0% to 0.1% weighted). In unweighted analyses, those who had high N/S coverage and were on OST, regardless of paraphernalia coverage, had lower odds of recent infection relative to those on the lowest coverage of interventions (AOR 0.28, 95% CI 0.08–0.98, *P* = 0.046 and AOR 0.28, 95% CI 0.08–0.97, *P* = 0.044). This finding was similar in the weighted analyses (AOR_w_ 0.02, 95% CI 0.01–0.09 and AOR_w_ 0.07, 95% CI 0.01–0.35 for those with low and high paraphernalia coverage, respectively). Most of the confidence intervals for the AORs of the different intervention coverage groups overlapped, with the exception of the ‘low N/S, low para, OST’ group (AOR_w_ 0.58, 95% CI 0.25–1.34) and the ‘high N/S, low para, OST’ group (AOR_w_ 0.02, 95% CI 0.01–0.09) in weighted analyses.

### New infections and infections averted

The estimated number of new infections per year declined from 1063 (95% CI 591–1682) in 2008 to 566 (95% CI 205–1039) in 2012 (Table 6). With regard to new chronic infections, these have potentially declined from 787 (95% CI 441–1248) in 2008 to 419 (95% CI 152–774) in 2012. We estimate that approximately 1,400 new infections and 1,000 new chronic infections may have been averted during 2008–2012.

**Table 6:** Estimated number of new infections and new chronic infections per calendar year based on derived incidence rates^a^.

	Estimated incidence per 100 PY (95% CI)	Proportion anti-HCV negative (95% CI)	No. new infections (95% CI)	No. new chronic infections^b^ (95% CI)
2008	13.6 (8.1–20.1)	0.48 (0.46–0.50)	1063 (591–1682)	787 (441–1248)
2009	13.6 (8.1–20.1)	0.48 (0.46–0.50)	1063 (591–1682)	787 (441–1248)
2010	9.6 (5.1–15.5)	0.45 (0.43–0.47)	697 (336–1240)	516 (251–908)
2011	7.3 (3.0–12.9)	0.47 (0.44–0.49)	566 (205–1039)	419 (152–774)
2012	7.3 (3.0–12.9)	0.47 (0.44–0.49)	566 (205–1039)	419 (152–774)

CI =  confidence interval; PY =  person-years.

a Number of PWID assumed to be stable during 2008–2012 at 16,000 (95%CI 11,782–20,334).

b 26% (95% CI 22–29%) assumed to clear acute infection [26].

## Discussion

We have observed a decline in HCV incidence among PWID during a period of development in harm reduction services in Scotland. This finding is corroborated by a similar trend observed in prevalence of HCV among recent initiates to injecting, which can be considered a proxy for incidence. A number of factors are likely to have contributed to the declining incidence of HCV: identifying the contributions of individual interventions is the challenge.

We applied a framework approach to bring together evidence for all of the steps and relationships on the pathways from interventions to outcome. Considering first the ecological/group-level analyses: in terms of the provision of interventions, it would appear that the largest change was the increase in distribution of filters and spoons. The contemporaneous increase in the self-reported uptake of filters and spoons over the three surveys, in terms of both numbers of items and coverage, would appear to reflect this change in provision. Furthermore, the increases in uptake of paraphernalia were mirrored by significant declines in the self-reported sharing of these items over the period, as would be expected.

By contrast, the finding of an initial decline in the self-reported numbers of sterile N/S obtained by individuals is apparently inconsistent with the relatively stable numbers of needles/syringes distributed. However, the decrease in self-reported numbers of needles/syringes obtained by respondents is offset by declines in the frequency of injecting, such that the proportion reporting high N/S coverage across the three surveys remained more or less stable (based on self-report), suggesting that the finding of less uptake is explained by less need. By contrast, the alternative measure of coverage (based on service provision) showed an increase from approximately half to three quarters of PWID with adequate coverage. Given this was based on stable numbers of N/S distributed, it was again the declining frequency of injecting that caused the change (from a mean of approximately 550 injections per PWID down to 400 in 2011–12). The observed reduction in reported N/S sharing is perhaps more consistent with increasing N/S coverage. Otherwise, the decline in sharing might be explained by a potential improvement in the quantity/quality of education that is being provided during injecting equipment transactions, as recommended in the national guidelines [11], leading to a greater awareness of the risks of injection. Thus, if people have usually been obtaining sufficient needles/syringes for their injections but not using all of them, coverage could feasibly stay the same while sharing goes down. A similar enigma was observed for water: self-reported sharing of water declined despite distribution of sterile water ampoules not having changed [19].

The increase in self-reported uptake of OST among survey participants also occurred contemporaneously with more or less stable dispensation of methadone prescriptions. Although we do not have information on how the PWID population has changed over the period examined, speculatively, if the PWID population had decreased, this might explain how the same number of prescriptions could translate into an increase in the uptake of OST. The increased uptake of OST was mirrored by a decrease in the self-reported frequency of injecting across the three surveys.

From an ecological perspective, we have a situation whereby data on the provision of interventions, uptake of interventions, and corresponding risk behaviour usually, but not always, paint a consistent picture. Thus, while ecological analyses can give us an overview of trends, they generally do not provide insight into the relationships and, moreover, can highlight discrepancies in the findings that need to be further investigated using individual-level analyses.

In the multivariable analyses for the three interventions independently, both N/S and paraphernalia coverage were associated with reduced risk of recent HCV infection, although it is notable that these models were not adjusted for the other respective interventions.

The unweighted associations represent risks per individual, and individuals may have injected few or many times. Several of the associations that were not significant in unweighted analyses became significant in weighted analyses, and indeed many of the effect sizes also changed, indicating that the frequency of injecting of individuals in particular intervention/outcome groups is potentially obscuring some of the intervention impact in the unweighted models. For example, the AOR for high N/S coverage reduced from 0.39 to 0.14; this was because those with low N/S coverage – in particular, those with incident infection – reported injecting more frequently, whereas those with high N/S coverage – also the incident infections in particular – reported injecting less frequently. The weighting therefore had the effect of amplifying the difference in the proportion of recent infections between the low and high coverage groups, which moved the effect size further from null.

We did not observe a significant association between uptake of OST alone and incident HCV. It is possible that we had insufficient power to detect an effect, given that OST coverage is so high in our study population. Another consideration is that the association between OST and HCV is an indirect one, since OST affects frequency of injecting, which is not in itself a mode of HCV transmission (as is sharing needles/syringes). The theory is that higher OST uptake should reduce HCV risk, by reducing the frequency of injecting and, consequently, the probability of sharing injecting equipment. Our analyses confirmed that OST was associated with a reduced frequency of injecting, and that lower frequency of injecting was associated with less sharing of all types of equipment. From the unadjusted incidence, it appeared that OST had a larger effect among those on low coverage of N/S and paraphernalia, which would be expected, given that the impact of injecting frequency on HCV transmission would be augmented if insufficient sterile equipment was being used.

Despite no effect of OST alone, it was associated with recent HCV in combination with N/S: being on OST and having high coverage N/S was associated with a greater reduction in risk of recent HCV (in weighted analyses, a 95% reduction in risk) as compared with either of the separate intervention effects, although our analysis was underpowered to demonstrate statistical significance.

In the combined interventions model stratified for paraphernalia, there was little difference in effect size between the groups with low and high paraphernalia coverage when the other interventions wereunchanged.Reasons for the lack of association between paraphernalia coverage and recent HCV could be:that uptake of paraphernalia is associated with a lower reduction in sharing as compared with uptake of needles/syringes (i.e. although PWID are obtaining paraphernalia from services, they are not using all of them); and/or that there is a lower risk of HCV transmission associated with sharing paraphernalia, such that a reduction in sharing paraphernalia might have less of an effect on transmission as compared with a reduction in needle/syringe sharing.

Our findings regarding paraphernalia coverage do not necessarily mean that providing paraphernalia is ineffective with regard to preventing HCV transmission. To evaluate the impact of sterile paraphernalia, one would ideally compare the incidence between high and low paraphernalia coverage groups solely among those with low N/S coverage. This was not possible here because there were too few people in these groups (approximately 50 in total), due to the fact that uptake of paraphernalia generally goes hand-in-hand with uptake of needles/syringes. It is possible that our analyses are underpowered to detect an effect and that pooling further sweeps of NESI would allow us to examine this with a larger sample size. A further consideration is that, for sharing paraphernalia to pose a risk of HCV transmission, it must first become contaminated with blood from a used needle/syringe. Thus, the risk from sharing paraphernalia is dependent on the rates of reuse or sharing of needles/syringes, both of which have declined over the period of study. If the rates of needle/syringe sharing or reuse in Scotland were to rise, the availability of sterile paraphernalia might become more critical in preventing HCV transmission.

Although we have observed encouraging signs in the direction of HCV incidence, we have not seen any changes in HCV prevalence. A mathematical modelling study has suggested that high coverage levels of both OST and NSP would need to be sustained for a 15-year period in order to reduce prevalence by a third [9]. Thus, it may be the case that it will take many years of a sustained reduction in HCV transmission before any changes in prevalence are observed.

### Comparability of our results

Scotland is one of few countries world-wide to have a surveillance system, with national coverage, that generates serial measures of HCV incidence. While one-off studies have been done to measure incidence in regional populations of PWID in the UK [23], [27], [28], it is not known whether the decline in HCV incidence observed here has been replicated elsewhere in the UK. Internationally, others have reported reductions in HCV incidence among PWID; however, these have tended to be over very long periods of time (often decades), restricted to regional populations, involving smaller sample sizes than ours, and/or involving lower coverage of interventions as compared with Scotland [29], [30]. Furthermore, it is notable that the latter declines in HCV occurred within the context of major shifts away from heroin and/or injecting [29], [31]. Although we have observed a reduction in frequency of injecting (related to increased prescribed methadone), this is still in context of a country with major heroin injecting problem [25]. Thus, other countries with persistent injecting populations can draw inferences on the potential impact of high coverage IEP and OST from our findings.

Our findings regarding OST are in contrast to other reports in the literature, which have found a significant association between OST uptake and HCV. In a synthesis of UK data, Turner et al. [7] found that receiving OST was associated with an approximately 60% reduction in odds of incident HCV (AOR 0.41, 95%CI: 0.21–0.82). Turner et al. also found that high N/S coverage was associated with a reduced risk of recent HCV (AOR 0.48, 95% CI: 0.25–0.93), similar to our adjusted (unweighted) effect size.

Finally, Turner et al. determined that ‘full’ harm reduction (i.e. on OST plus high N/S coverage) reduced the odds of new HCV infection by nearly 80% (AOR = 0.21, 95% CI: 0.08–0.52), which is similar to our (unweighted) AOR for combined coverage.

### Strengths and limitations

Whereas most analyses take either an ecological or an individual-level approach, in this study we have attempted to consider both types of evidence in conjunction. Constructing a coherent narrative from the ecological data can be difficult, particularly when trying to reconcile data from different sources, such as service provision and selected population samples. The limitations of drawing inferences solely from ecological analysis are also apparent from this analysis; on the other hand, considering just the individual-level evidence does not give us the overview of trends in provision, uptake, risk behaviour, and HCV incidence. Although these types of evidence will never provide the same level of confidence, in terms of a causal association between intervention(s) and outcome, as a randomised controlled trial, the triangulation of evidence generated by different study designs is understood to increase confidence [17]. In the ecological analyses, other contemporaneous interventions or factors could potentially have been responsible for some of the observed changes; the individual analyses provide validation for some of these associations. For example, that the uptake of interventions is associated with reduced risk behaviour at the individual-level means we can therefore be more confident that the changes in risk behaviour observed were due to the provision of the interventions. There are nevertheless factors that were outside the scope of these analyses that could have had an impact on HCV incidence; for example, HCV antiviral treatment. However, treatment rates remain low in this population group and so treatment, alone, is unlikely to account fully for the reduced HCV incidence. Nevertheless, the potential impact of HCV treatment has been demonstrated in mathematical models [32], [33] and it is possible that, in combination with IEP and OST, it contributed to the reduction in HCV transmission observed here.

Selection bias can be an issue in non-randomised studies. The NESI studies recruited participants at services that provide sterile injecting equipment (and often dispense methadone also). This approach may have excluded ‘high risk’ PWID who are not in contact with services,which may have resulted in an underestimate of HCV incidence (assumeingthat those not attending services are at greater risk of HCV infection) and an underestimate of intervention impact (assuming those not attending services would have contributed to the group with recent infection and low coverage of interventions). However, that we have demonstrated a decline in incidence among those attending services is nevertheless and important finding in itself, and is indicative that these services are having an impact among attendees. There is also the issue of the comparability of consecutive NESI surveys. While we attempted to maximise consistency in recruitment across surveys, it was not always possible to recruit the from same services year-on-year. There are indications that the PWID population in Scotland is an ageing cohort: we have observed an increase in the average age and time since onset of injecting over the years. That older PWID are less likely to engage in risk behaviour may explain some of the downward trends in risk behaviour and incidence. However, we do not believe that this would be sufficient to explain the sharpness of the downward trends observed, and furthermore the finding of a declining prevalence among those who had commenced injecting with the previous year provides evidence that this decline in HCV is also occurring among newer PWID. In addition, the multivariate models demonstrated associations with harm reduction interventions even after adjustment for time since onset of injecting, suggesting that the latter does not fully account for the observed changes in HCV incidence.

Despite our large sample size, we detected only just over 50 recent infections in the pooled analysis: thus, the lack of statistical significance in some cases – particularly when examining interventions classified into multiple strata – may have been a result of lack of power. In a scenario of declining HCV incidence, increasingly large sample sizes will be required to detect increasingly small numbers of recent infections.

The derived incidence estimates are reliant on an accurate figure for the duration of the pre-seroconversion window period, around which there is uncertainty [24]. We will not have captured persons who had recently seroconverted at the time of interview; however, the incidence calculation takes this underestimation into account. We would generally not detect re-infections: people who hadbeen infected in the past would have HCV antibody and would have been classed as prevalent infections. By contrast, some of the recent infections may have been individuals who cleared infection and lost antibodies (i) over time [34] or (ii) due to HIV-coinfection [35]. However, these are unlikely to account for many recent infections since (i) the majority of recent infections were among PWID with less than five years injecting and (ii) HIV prevalence is very low in this population [36].

In relation to OST, one of the limitations of this study is that it is not specifically designed to measure the impact of this intervention. Questions on methadone dosage have not been asked consistently across the surveys, therefore it was not possible to examine the association between dosage and recent HCV; moreover, dosage itself is a problematic measure, as what constitutes an adequate dosage can vary greatly from person to person. At the provision level, the absence of available data on persons receiving methadone mixture meant that numbers of methadone mixture prescriptions were presented instead: these could be misleading because a single prescription can indicate single or multiple dose(s). Assuming that prescribing practices have not changed drastically over the period of study, then the observed trends are still likely to be valid.

Finally, the self-reported nature of the risk behaviours and uptake of harm reduction interventions is a limitation. While the self-reported data likely contains an element of inaccuracy due to difficulty with respondents’ ability to recall events, the consistency of the associations between self-reported behaviour and biological markers (both HCV prevalence and incidence) is high, lending credence to the validity of the data.

### Conclusions

These data provide evidence of a downward trend in HCV incidence among PWID in Scotland. The two different approaches used in this analysis strengthen the inference that the changes in HCV incidence are attributable to harm reduction interventions – particularly N/S and OST combined. Future monitoring of PWID will be required to establish whether the downward direction in HCV transmission among PWID in Scotland is sustained.

## Supporting Information

Table S1 **Numbers of items of injecting equipment distributed and methadone mixture prescriptions dispensed in Scotland, by financial year, 2008/09 to 2010/11^a^.** ^a^Modified from Injecting Equipment Provision in Scotland Survey 2010/11 [19] and Drug Misuse Statistics Scotland 2011 [20]. ^b^Figures have been adjusted by the proportion of total services providing data for respective financial years.
(DOCX)

Table S2 **Data used to calculate IEP coverage (based on service provision data).** N/S =  needles/syringes; PWID =  people who inject drugs. ^a^Data correspond to financial years, i.e. 2008/09, 2010/11 and 2011/12.
(DOCX)

Table S3 **Univariable and multivariable models of the association between needle/syringe coverage and sharing needles/syringes (in the last 6 months), including covariates^a^.** N/S =  needles/syringes. ^a^Models are restricted to those who reported injecting in the last six months. ^b^Excessive is defined as >14 units/week for women and >21 units/week for men.
(DOCX)

Table S4 **Univariable and multivariable models of the association between spoon coverage and sharing spoons (in the last 6 months), including covariates^a^.** ^a^Models are restricted to those who reported injecting in the last six months. ^b^Excessive is defined as >14 units/week for women and >21 units/week for men.
(DOCX)

Table S5 **Univariable and multivariable models of the association between filter coverage and sharing filters (in the last 6 months), including covariates^a^.** ^a^Models are restricted to those who reported injecting in the last six months. ^b^Excessive is defined as >14 units/week for women and >21 units/week for men.
(DOCX)

Table S6 **Univariable and multivariable models of the association between OST and injecting daily or more frequently, including covariates^a^.** ^a^Models exclude individuals who reported not currently being on OST and also not injecting in the last six months.
(DOCX)

Table S7 **Univariable and multivariable models of the association between frequency of injecting (in the last 6 months) and recent HCV infection^a^.** ^a^Models exclude individuals who reported not currently being on OST and also not injecting in the last six months. ^b^Excessive is defined as >14 units/week for women and >21 units/week for men.
(DOCX)

Table S8 **Univariable and multivariable models of the association between frequency of injecting in the last 6 months and sharing needle/syringes in the last six months^a^.** ^a^Models exclude individuals who reported not currently being on OST and also not injecting in the last six months. ^b^Excessive is defined as >14 units/week for women and >21 units/week for men.
(DOCX)

Table S9 **Univariable and multivariable models of the association between frequency of injecting in the last 6 months and sharing spoons in the last six months^a^.** ^a^Models exclude individuals who reported not currently being on OST and also not injecting in the last six months. ^b^Excessive is defined as >14 units/week for women and >21 units/week for men.
(DOCX)

Table S10 **Univariable and multivariable models of the association between frequency of injecting in the last 6 months and sharing filters in the last six months^a^.** ^a^Models exclude individuals who reported not currently being on OST and also not injecting in the last six months. ^b^Excessive is defined as >14 units/week for women and >21 units/week for men.
(DOCX)
