# Supplementary material for: Rapid Decline in HCV Incidence among People Who Inject Drugs Associated with National Scale-Up in Coverage of a Combination of Harm Reduction Interventions
Source: PLoS One. 2014 Aug 11;9(8):e104515. doi: 10.1371/journal.pone.0104515 (PMC4128763; doi:10.1371/journal.pone.0104515)
Supplement: Table S1 — Numbers of items of injecting equipment distributed and methadone mixture prescriptions dispensed in Scotland, by financial year, 2008/09 to 2010/11a. aModified from Injecting Equipment Provision in Scotland Survey 2010/11 [19] and Drug Misuse Statistics Scotland 2011 [20]. bFigures have been adjusted by the proportion of total services providing data for respective financial years. (DOCX) [file pone.0104515.s001.docx]

**Table S1.** Numbers of items of injecting equipment distributed and methadone mixture prescriptions dispensed in Scotland, by financial year, 2008/09 to 2010/11^a^

|  | 2008/09 | 2009/10 | 2010/11 | 2011/12 | Factor increase between 2008/09 and 2009/10 | Factor increase between 2009/10 and 2010/11 | Factor increase between 2010/11 and 2011/12 |
| --- | --- | --- | --- | --- | --- | --- | --- |
| **Injecting equipment items distributed** | | |  |  |  |  |  |
| Needles/syringes^b^ | 4,736,700 | 4,699,600 | 4,626,700 | 4,722,500 | 0.99 | 0.98 | 1.02 |
| Filters | 355,872 | 2,224,259 | 2,500,147 | 2,534,289 | 6.25 | 1.12 | 1.01 |
| Water ampoules | 62,229 | 77,352 | 71,575 | 68,984 | 1.24 | 0.93 | 0.96 |
| Spoons | 508,515 | 2,142,740 | 2,438,381 | 2,527,480 | 4.21 | 1.14 | 1.04 |
| **Methadone mixture prescriptions dispensed** | | | |  |  |  |  |
|  | 493,767 | 510,063 | 534,674 | 515,897 | 1.03 | 1.05 | 0.96 |

^a^Modified from Injecting Equipment Provision in Scotland Survey 2010/11 [14] and Drug Misuse Statistics Scotland 2011 [15]

^b^Figures have been adjusted by the proportion of total services providing data for respective financial years
