# Supplementary material for: Rapid Decline in HCV Incidence among People Who Inject Drugs Associated with National Scale-Up in Coverage of a Combination of Harm Reduction Interventions
Source: PLoS One. 2014 Aug 11;9(8):e104515. doi: 10.1371/journal.pone.0104515 (PMC4128763; doi:10.1371/journal.pone.0104515)
Supplement: Table S2 — Data used to calculate IEP coverage (based on service provision data). N/S = needles/syringes; PWID = people who inject drugs. aData correspond to financial years, i.e. 2008/09, 2010/11 and 2011/12. (DOCX) [file pone.0104515.s002.docx]

**Table S2.** Data used to calculate IEP coverage (based on service provision data)

|  | **2008-09** | **2010** | **2011-12** |
| --- | --- | --- | --- |
| No. N/S distributed^a^ | 4,736,700 | 4,626,700 | 4,722,500 |
| No. spoons distributed^a^ | 508,515 | 2,438,381 | 2,527,480 |
| No. filters distributed^a^ | 355,872 | 2,500,147 | 2,534,289 |
| Mean no. injections per PWID (12 mths) | 554 | 464 | 400 |
| No. of PWID | 16,000 | 16,000 | 16,000 |
| Total annual no. injections (mean) | 8,864,000 | 7,424,000 | 6,400,000 |
| N/S coverage (mean) | 53 | 62 | 74 |
| Spoon coverage (mean) | 6 | 33 | 39 |
| Filter coverage (mean) | 4 | 34 | 40 |

N/S = needles/syringes; PWID = people who inject drugs

^a^Data correspond to financial years, i.e. 2008/09, 2010/11 and 2011/12
