# Supplementary material for: Rapid Decline in HCV Incidence among People Who Inject Drugs Associated with National Scale-Up in Coverage of a Combination of Harm Reduction Interventions
Source: PLoS One. 2014 Aug 11;9(8):e104515. doi: 10.1371/journal.pone.0104515 (PMC4128763; doi:10.1371/journal.pone.0104515)
Supplement: Table S3 — Univariable and multivariable models of the association between needle/syringe coverage and sharing needles/syringes (in the last 6 months), including covariatesa. N/S = needles/syringes. aModels are restricted to those who reported injecting in the last six months. bExcessive is defined as >14 units/week for women and >21 units/week for men. (DOCX) [file pone.0104515.s003.docx]

**Table S3.** Univariable and multivariable models of the association between needle/syringe coverage and sharing needles/syringes (in the last 6 months), including covariates^a^

|  |  |  |  |  | Univariable | | | Multivariable (n=5,409) | | |
| --- | --- | --- | --- | --- | --- | --- | --- | --- | --- | --- |
|  |  | Total (N) | No. who shared N/S (n) | % (n/N) | OR | 95% CI | *P* value | AOR | 95% CI | *P* value |
| **N/S coverage** | **<100%** | **1274** | **227** | **18** | **1** |  |  | **1** |  |  |
|  | **100-199%** | **1913** | **234** | **12** | **0.64** | **0.53-0.78** | **<0.001** | **0.64** | **0.52-0.79** | **<0.001** |
|  | **≥200%** | **2264** | **195** | **9** | **0.44** | **0.35-0.53** | **<0.001** | **0.44** | **0.35-0.54** | **<0.001** |
| Survey | 2008-09 | 2046 | 310 | 15 | 1 |  |  | 1 |  |  |
|  | 2010 | 2063 | 229 | 11 | 0.70 | 0.58-0.84 | <0.001 | 0.77 | 0.64-0.93 | 0.008 |
|  | 2011-12 | 1379 | 120 | 9 | 0.53 | 0.43-0.67 | <0.001 | 0.59 | 0.47-0.74 | <0.001 |
| Gender | Male | 4035 | 453 | 11 | 1 |  |  | 1 |  |  |
|  | Female | 1430 | 201 | 14 | 1.29 | 1.08-1.55 | 0.005 | 1.35 | 1.12-1.63 | 0.002 |
| Homeless in last 6 months | No | 4067 | 425 | 10 | 1 |  |  | 1 |  |  |
|  | Yes | 1413 | 231 | 16 | 1.68 | 1.41-1.99 | <0.001 | 1.34 | 1.12-1.61 | 0.002 |
| Injected stimulant in last 6 months | No | 4552 | 495 | 11 | 1 |  |  | 1 |  |  |
|  | Yes | 935 | 164 | 18 | 1.74 | 1.44-2.11 | <0.001 | 1.58 | 1.29-1.94 | <0.001 |
| Alcohol consumption in the last 12 months^b^ | Not excessive | 4047 | 414 | 10 | 1 |  |  | 1 |  |  |
|  | Excessive | 1412 | 239 | 17 | 1.79 | 1.51-2.12 | <0.001 | 1.68 | 1.40-2.01 | <0.001 |
| Current OST | No | 1716 | 256 | 15 | 1 |  |  | 1 |  |  |
|  | Yes | 3771 | 403 | 11 | 0.68 | 0.58-0.81 | <0.001 | 0.80 | 0.67-0.96 | 0.014 |
| Age (years) | <25 | 761 | 147 | 19 | 1 |  |  | 1 |  |  |
|  | 25+ | 4724 | 512 | 11 | 0.51 | 0.42-0.62 | <0.001 | 0.59 | 0.47-0.73 | <0.001 |

N/S = needles/syringes

^a^Models are restricted to those who reported injecting in the last six months

^b^Excessive is defined as >14 units/week for women and >21 units/week for men
