# Supplementary material for: Rapid Decline in HCV Incidence among People Who Inject Drugs Associated with National Scale-Up in Coverage of a Combination of Harm Reduction Interventions
Source: PLoS One. 2014 Aug 11;9(8):e104515. doi: 10.1371/journal.pone.0104515 (PMC4128763; doi:10.1371/journal.pone.0104515)
Supplement: Table S4 — Univariable and multivariable models of the association between spoon coverage and sharing spoons (in the last 6 months), including covariatesa. aModels are restricted to those who reported injecting in the last six months. bExcessive is defined as >14 units/week for women and >21 units/week for men. (DOCX) [file pone.0104515.s004.docx]

**Table S4.** Univariable and multivariable models of the association between spoon coverage and sharing spoons (in the last 6 months), including covariates^a^

|  |  |  |  |  | Univariable | | | Multivariable (n=5,419) | | |
| --- | --- | --- | --- | --- | --- | --- | --- | --- | --- | --- |
|  |  | Total (N) | No. who shared spoons (n) | % (n/N) | OR | 95% CI | *P* value | AOR | 95% CI | *P* value |
| **Spoon coverage** | **<100%** | **2945** | **1165** | **40** | **1** |  |  | **1** |  |  |
|  | **100-199%** | **1196** | **329** | **28** | **0.58** | **0.50-0.67** | **<0.001** | **0.72** | **0.62-0.85** | **<0.001** |
|  | **≥200%** | **1320** | **326** | **25** | **0.50** | **0.43-0.58** | **<0.001** | **0.63** | **0.54-0.74** | **<0.001** |
| Survey | 2008-09 | 2052 | 868 | 42 | 1 |  |  | 1 |  |  |
|  | 2010 | 2063 | 694 | 34 | 0.69 | 0.61-0.79 | <0.001 | 0.87 | 0.76-1.00 | 0.055 |
|  | 2011-12 | 1380 | 266 | 19 | 0.33 | 0.28-0.38 | <0.001 | 0.42 | 0.35-0.50 | <0.001 |
| Gender | Male | 4035 | 1281 | 32 | 1 |  |  | 1 |  |  |
|  | Female | 1437 | 538 | 37 | 1.29 | 1.14-1.46 | <0.001 | 1.35 | 1.18-1.55 | <0.001 |
| Homeless in last 6 months | No | 4069 | 1217 | 30 | 1 |  |  | 1 |  |  |
|  | Yes | 1418 | 608 | 43 | 1.76 | 1.55-1.99 | <0.001 | 1.50 | 1.32-1.72 | <0.001 |
| Injected stimulant in last 6 months | No | 4557 | 1405 | 31 | 1 |  |  | 1 |  |  |
|  | Yes | 937 | 423 | 45 | 1.85 | 1.60-2.13 | <0.001 | 1.63 | 1.40-1.90 | <0.001 |
| Alcohol consumption in the last 12 months^b^ | Not excessive | 4051 | 1190 | 29 | 1 |  |  | 1 |  |  |
|  | Excessive | 1415 | 628 | 44 | 1.92 | 1.69-2.17 | <0.001 | 1.79 | 1.57-2.05 | <0.001 |
| Current OST | No | 1717 | 636 | 37 | 1 |  |  | 1 |  |  |
|  | Yes | 3777 | 1192 | 32 | 0.78 | 0.70-0.88 | <0.001 | 0.90 | 0.79-1.02 | 0.094 |
| Age (years) | <25 | 762 | 336 | 44 | 1 |  |  | 1 |  |  |
|  | 25+ | 4730 | 1491 | 32 | 0.58 | 0.50-0.68 | <0.001 | 0.66 | 0.56-0.78 | <0.001 |

^a^Models are restricted to those who reported injecting in the last six months

^b^Excessive is defined as >14 units/week for women and >21 units/week for men
