# Supplementary material for: Rapid Decline in HCV Incidence among People Who Inject Drugs Associated with National Scale-Up in Coverage of a Combination of Harm Reduction Interventions
Source: PLoS One. 2014 Aug 11;9(8):e104515. doi: 10.1371/journal.pone.0104515 (PMC4128763; doi:10.1371/journal.pone.0104515)
Supplement: Table S5 — Univariable and multivariable models of the association between filter coverage and sharing filters (in the last 6 months), including covariatesa. aModels are restricted to those who reported injecting in the last six months. bExcessive is defined as >14 units/week for women and >21 units/week for men. (DOCX) [file pone.0104515.s005.docx]

**Table S5.** Univariable and multivariable models of the association between filter coverage and sharing filters (in the last 6 months), including covariates^a^

|  |  |  |  |  | Univariable | | | Multivariable (n=5,413) | | |
| --- | --- | --- | --- | --- | --- | --- | --- | --- | --- | --- |
|  |  | Total (N) | No. who shared filters (n) | % (n/N) | OR | 95% CI | *P* value | AOR | 95% CI | *P* value |
| **Filter coverage** | **<100%** | **2882** | **871** | **30** | **1** |  |  | **1** |  |  |
|  | **100-199%** | **1235** | **306** | **25** | **0.76** | **0.65-0.89** | **<0.001** | **0.90** | **0.76-1.06** | **0.211** |
|  | **≥200%** | **1344** | **297** | **22** | **0.66** | **0.56-0.76** | **<0.001** | **0.81** | **0.69-0.95** | **0.011** |
| Survey | 2008-09 | 2055 | 671 | 33 | 1 |  |  | 1 |  |  |
|  | 2010 | 2063 | 576 | 28 | 0.80 | 0.70-0.91 | 0.001 | 0.94 | 0.81-1.09 | 0.390 |
|  | 2011-12 | 1380 | 233 | 17 | 0.42 | 0.35-0.50 | <0.001 | 0.50 | 0.42-0.60 | <0.001 |
| Gender | Male | 4037 | 1036 | 26 | 1 |  |  | 1 |  |  |
|  | Female | 1438 | 434 | 30 | 1.25 | 1.10-1.43 | 0.001 | 1.40 | 1.21-1.62 | <0.001 |
| Homeless in last 6 months | No | 4073 | 967 | 24 | 1 |  |  | 1 |  |  |
|  | Yes | 1417 | 511 | 36 | 1.81 | 1.59-2.06 | <0.001 | 1.53 | 1.34-1.76 | <0.001 |
| Injected stimulant in last 6 months | No | 4558 | 1124 | 25 | 1 |  |  | 1 |  |  |
|  | Yes | 939 | 356 | 38 | 1.87 | 1.61-2.16 | <0.001 | 1.67 | 1.43-1.95 | <0.001 |
| Imprisoned | Never | 2188 | 540 | 25 | 1 |  |  | 1 |  |  |
|  | Ever | 3302 | 938 | 28 | 1.21 | 1.07-1.37 | 0.002 | 1.21 | 1.06-1.38 | 0.006 |
| Alcohol consumption in the last 12 months^b^ | Not excessive | 4052 | 958 | 24 | 1 |  |  | 1 |  |  |
|  | Excessive | 1417 | 511 | 36 | 1.82 | 1.60-2.08 | <0.001 | 1.65 | 1.44-1.89 | <0.001 |
| Current OST | No | 1718 | 529 | 31 | 1 |  |  | 1 |  |  |
|  | Yes | 3779 | 951 | 25 | 0.76 | 0.67-0.86 | <0.001 | 0.81 | 0.71-0.93 | 0.002 |
| Age (years) | <25 | 763 | 258 | 34 | 1 |  |  | 1 |  |  |
|  | 25+ | 4732 | 1221 | 26 | 0.68 | 0.58-0.80 | <0.001 | 0.79 | 0.66-0.94 | 0.008 |

^a^Models are restricted to those who reported injecting in the last six months

^b^Excessive is defined as >14 units/week for women and >21 units/week for men
