# Supplementary material for: Rapid Decline in HCV Incidence among People Who Inject Drugs Associated with National Scale-Up in Coverage of a Combination of Harm Reduction Interventions
Source: PLoS One. 2014 Aug 11;9(8):e104515. doi: 10.1371/journal.pone.0104515 (PMC4128763; doi:10.1371/journal.pone.0104515)
Supplement: Table S6 — Univariable and multivariable models of the association between OST and injecting daily or more frequently, including covariatesa. aModels exclude individuals who reported not currently being on OST and also not injecting in the last six months. (DOCX) [file pone.0104515.s006.docx]

**Table S6. Univariable and multivariable models of the association between OST and injecting daily or more frequently, including covariates.^a^**

|  |  |  |  |  | Univariable | | | Multivariable (n=6,776) | | |
| --- | --- | --- | --- | --- | --- | --- | --- | --- | --- | --- |
|  |  | Total (N) | No. injected at least daily (n) | % (n/N) | OR | 95% CI | *P* value | AOR | 95% CI | *P* value |
| **Current OST** | **No** | **1724** | **1263** | **73** | **1** |  |  | **1** |  |  |
|  | **Yes** | **5104** | **1869** | **37** | **0.21** | **0.19-0.24** | **<0.001** | **0.22** | **0.20-0.25** | **<0.001** |
| Survey | 2008-09 | 2552 | 1295 | 51 | 1 |  |  | 1 |  |  |
|  | 2010 | 2666 | 1123 | 42 | 0.71 | 0.63-0.79 | <0.001 | 0.84 | 0.74-0.94 | 0.003 |
|  | 2011-12 | 1611 | 715 | 44 | 0.78 | 0.68-0.88 | <0.001 | 0.89 | 0.78-1.02 | 0.088 |
| Homeless in last 6 months | No | 5214 | 2209 | 42 | 1 |  |  | 1 |  |  |
|  | Yes | 1607 | 920 | 57 | 1.82 | 1.63-2.04 | <0.001 | 1.50 | 1.33-1.69 | <0.001 |
| Injected stimulant in last 6 months | No | 5887 | 2527 | 43 | 1 |  |  | 1 |  |  |
|  | Yes | 941 | 606 | 64 | 2.41 | 2.09-2.78 | <0.001 | 2.22 | 1.90-2.58 | <0.001 |
| Imprisoned | Never | 2722 | 1181 | 43 | 1 |  |  | 1 |  |  |
|  | Ever | 4094 | 1946 | 48 | 1.18 | 1.07-1.30 | 0.001 | 1.27 | 1.14-1.41 | <0.001 |
| Age (years) | <25 | 836 | 496 | 59 | 1 |  |  | 1 |  |  |
|  | 25+ | 5988 | 2636 | 44 | 0.54 | 0.47-0.63 | <0.001 | 0.70 | 0.60-0.83 | <0.001 |

^a^Models exclude individuals who reported not currently being on OST and also not injecting in the last six months
