# Supplementary material for: Rapid Decline in HCV Incidence among People Who Inject Drugs Associated with National Scale-Up in Coverage of a Combination of Harm Reduction Interventions
Source: PLoS One. 2014 Aug 11;9(8):e104515. doi: 10.1371/journal.pone.0104515 (PMC4128763; doi:10.1371/journal.pone.0104515)
Supplement: Table S7 — Univariable and multivariable models of the association between frequency of injecting (in the last 6 months) and recent HCV infectiona. aModels exclude individuals who reported not currently being on OST and also not injecting in the last six months. bExcessive is defined as >14 units/week for women and >21 units/week for men. (DOCX) [file pone.0104515.s007.docx]

**Table S7. Univariable and multivariable models of the association between frequency of injecting (in the last 6 months) and recent HCV infection.^a^**

|  |  |  |  |  | Univariable | | | Multivariable (n=2,999) | | |
| --- | --- | --- | --- | --- | --- | --- | --- | --- | --- | --- |
|  |  | Total (N) | No. recent HCV infections (n) | % (n/N) | OR | 95% CI | *P* value | AOR | 95% CI | *P* value |
| **Injected daily or more frequently in the last 6 months** | **No** | **1659** | **19** | **1.1** | **1** |  |  | **1** |  |  |
|  | **Yes** | **1365** | **31** | **2.3** | **2.01** | **1.13-3.57** | **0.018** | **1.45** | **0.80-2.63** | **0.218** |
| Survey | 2008/09 | 1116 | 23 | 2.1 | 1 |  |  | 1 |  |  |
|  | 2010 | 1130 | 19 | 1.7 | 0.81 | 0.44-1.50 | 0.507 | 0.92 | 0.49-1.72 | 0.795 |
|  | 2011/12 | 778 | 8 | 1.0 | 0.49 | 0.22-1.11 | 0.088 | 0.56 | 0.25-1.27 | 0.166 |
| Homeless in last 6 months | No | 2392 | 25 | 1.0 | 1 |  |  | 1 |  |  |
|  | Yes | 629 | 25 | 4.0 | 3.92 | 2.24-6.87 | <0.001 | 3.02 | 1.70-5.36 | <0.001 |
| Injected stimulant in last 6 months | No | 2731 | 39 | 1.4 | 1 |  |  | 1 |  |  |
|  | Yes | 293 | 11 | 3.8 | 2.69 | 1.36-5.32 | 0.004 | 2.26 | 1.11-4.58 | 0.024 |
| Time since onset of injecting | <5 years | 1186 | 32 | 2.7 | 1 |  |  | 1 |  |  |
|  | ≥5 years | 1826 | 18 | 1.0 | 0.36 | 0.20-0.64 | 0.001 | 0.39 | 0.22-0.71 | 0.002 |
| Alcohol consumption in last 12 months^b^ | Not excessive | 2383 | 31 | 1.3 | 1 |  |  | 1 |  |  |
|  | Excessive | 631 | 19 | 3.0 | 2.36 | 1.32-4.20 | 0.004 | 1.93 | 1.07-3.50 | 0.030 |

^a^Models exclude individuals who reported not currently being on OST and also not injecting in the last six months

^b^Excessive is defined as >14 units/week for women and >21 units/week for men
