# Supplementary material for: Rapid Decline in HCV Incidence among People Who Inject Drugs Associated with National Scale-Up in Coverage of a Combination of Harm Reduction Interventions
Source: PLoS One. 2014 Aug 11;9(8):e104515. doi: 10.1371/journal.pone.0104515 (PMC4128763; doi:10.1371/journal.pone.0104515)
Supplement: Table S8 — Univariable and multivariable models of the association between frequency of injecting in the last 6 months and sharing needle/syringes in the last six monthsa. aModels exclude individuals who reported not currently being on OST and also not injecting in the last six months. bExcessive is defined as >14 units/week for women and >21 units/week for men. (DOCX) [file pone.0104515.s008.docx]

**Table S8. Univariable and multivariable models of the association between frequency of injecting in the last 6 months and sharing needle/syringes in the last six months.^a^**

|  |  |  |  |  | Univariable | | | Multivariable (n=6,746) | | |
| --- | --- | --- | --- | --- | --- | --- | --- | --- | --- | --- |
|  |  | Total (N) | No. who shared N/S (n) | % (n/N) | OR | 95% CI | *P* value | AOR | 95% CI | *P* value |
| **Injected daily or more frequently in the last 6 months** | **No** | **3687** | **180** | **4.9** | **1** |  |  | **1** |  |  |
|  | **Yes** | **3119** | **479** | **15.4** | **3.54** | **2.96-4.23** | **<0.001** | **3.04** | **2.53-3.66** | **<0.001** |
| Survey | 2008/09 | 2537 | 310 | 12.2 | 1 |  |  | 1 |  |  |
|  | 2010 | 2663 | 229 | 8.6 | 0.67 | 0.56-0.81 | <0.001 | 0.80 | 0.66-0.97 | 0.020 |
|  | 2011/12 | 1606 | 120 | 7.5 | 0.58 | 0.47-0.72 | <0.001 | 0.68 | 0.54-0.85 | 0.001 |
| Gender | Male | 4903 | 453 | 9.2 | 1 |  |  | 1 |  |  |
|  | Female | 1879 | 201 | 10.7 | 1.18 | 0.99-1.40 | 0.069 | 1.21 | 1.01-1.46 | 0.042 |
| Homeless in last 6 months | No | 5197 | 425 | 8.2 | 1 |  |  | 1 |  |  |
|  | Yes | 1601 | 231 | 14.4 | 1.89 | 1.60-2.25 | <0.001 | 1.36 | 1.14-1.63 | 0.001 |
| Injected stimulant in last 6 months | No | 5870 | 495 | 8.4 | 1 |  |  | 1 |  |  |
|  | Yes | 935 | 164 | 17.5 | 2.31 | 1.91-2.80 | <0.001 | 1.75 | 1.42-2.14 | <0.001 |
| Time since onset of injecting | <5 years | 1678 | 211 | 12.6 | 1 |  |  | 1 |  |  |
|  | ≥5 years | 5107 | 447 | 8.8 | 0.67 | 0.56-0.79 | <0.001 | 0.80 | 0.66-0.98 | 0.031 |
| Alcohol consumption in last 12 months^b^ | Not excessive | 5057 | 414 | 8.2 | 1 |  |  | 1 |  |  |
|  | Excessive | 1719 | 239 | 13.9 | 1.81 | 1.53-2.15 | <0.001 | 1.75 | 1.46-2.09 | <0.001 |
| Age (years) | <25 | 833 | 147 | 17.6 | 1 |  |  | 1 |  |  |
|  | 25+ | 5968 | 512 | 8.6 | 0.44 | 0.36-0.54 | <0.001 | 0.58 | 0.46-0.73 | <0.001 |

^a^Models exclude individuals who reported not currently being on OST and also not injecting in the last six months

^b^Excessive is defined as >14 units/week for women and >21 units/week for men
