# Supplementary material for: Rapid Decline in HCV Incidence among People Who Inject Drugs Associated with National Scale-Up in Coverage of a Combination of Harm Reduction Interventions
Source: PLoS One. 2014 Aug 11;9(8):e104515. doi: 10.1371/journal.pone.0104515 (PMC4128763; doi:10.1371/journal.pone.0104515)
Supplement: Table S10 — Univariable and multivariable models of the association between frequency of injecting in the last 6 months and sharing filters in the last six monthsa. aModels exclude individuals who reported not currently being on OST and also not injecting in the last six months. bExcessive is defined as >14 units/week for women and >21 units/week for men. (DOCX) [file pone.0104515.s010.docx]

**Table S10. Univariable and multivariable models of the association between frequency of injecting in the last 6 months and sharing filters in the last six months.^a^**

|  |  | Total (N) |  | % (n/N) | Univariable | | | Multivariable (n=6,772) | | |
| --- | --- | --- | --- | --- | --- | --- | --- | --- | --- | --- |
|  |  |  | No. who shared filters (n) |  | OR | 95% CI | *P* value | AOR | 95% CI | *P* value |
| **Injected daily or more frequently in the last 6 months** | **No** | **3689** | **484** | **13.1** | **1** |  |  | **1** |  |  |
|  | **Yes** | **3127** | **996** | **31.9** | **3.10** | **2.74-3.50** | **<0.001** | **2.77** | **2.44-3.14** | **<0.001** |
| Survey | 2008/09 | 2546 | 671 | 26.4 | 1 |  |  | 1 |  |  |
|  | 2010 | 2663 | 576 | 21.6 | 0.77 | 0.68-0.88 | <0.001 | 0.93 | 0.81-1.07 | 0.308 |
|  | 2011/12 | 1607 | 233 | 14.5 | 0.47 | 0.40-0.56 | <0.001 | 0.54 | 0.45-0.64 | <0.001 |
| Gender | Male | 4905 | 1036 | 21.1 | 1 |  |  | 1 |  |  |
|  | Female | 1887 | 434 | 23.0 | 1.12 | 0.98-1.27 | 0.092 | 1.22 | 1.07-1.40 | 0.004 |
| Homeless in last 6 months | No | 5203 | 967 | 18.6 | 1 |  |  | 1 |  |  |
|  | Yes | 1605 | 511 | 31.8 | 2.05 | 1.80-2.32 | <0.001 | 1.59 | 1.39-1.82 | <0.001 |
| Injected stimulant in last 6 months | No | 5876 | 1124 | 19.1 | 1 |  |  | 1 |  |  |
|  | Yes | 939 | 356 | 37.9 | 2.58 | 2.23-2.99 | <0.001 | 1.98 | 1.69-2.32 | <0.001 |
| Alcohol consumption in last 12 months^b^ | Not excessive | 5062 | 958 | 18.9 | 1 |  |  | 1 |  |  |
|  | Excessive | 1724 | 511 | 29.6 | 1.81 | 1.59-2.05 | <0.001 | 1.68 | 1.47-1.92 | <0.001 |
| Age (years) | <25 | 835 | 258 | 30.9 | 1 |  |  | 1 |  |  |
|  | 25+ | 5976 | 1221 | 20.4 | 0.57 | 0.49-0.67 | <0.001 | 0.72 | 0.61-0.86 | <0.001 |

^a^Models exclude individuals who reported not currently being on OST and also not injecting in the last six months

^b^Excessive is defined as >14 units/week for women and >21 units/week for men
